# Effectiveness of educational intervention among seropositive women about knowledge about HIV sexual transmission

**DOI:** 10.1590/0034-7167-2022-0371

**Published:** 2023-08-21

**Authors:** Regina de Souza Alves, Laelson Rochelle Milanês Sousa, Josely Pinto de Moura, Elucir Gir, Renata Karina Reis

**Affiliations:** I Universidade de São Paulo. Ribeirão Preto, São Paulo, Brazil.; II Universidade Federal do Piauí. Teresina, Piauí, Brazil.; III Universidade do Estado de Minas Gerais. Passos, Minas Gerais, Brazil.

**Keywords:** HIV, Women, Health Knowledge, Attitudes, Practice, Health Promotion, Information Technology, VIH, Mujeres, Conocimientos, Actitudes Y Práctica en Salud, Promoción de la Salud, Tecnología de la Información, HIV, Mulheres, Conhecimentos, Atitudes e Prática em Saúde, Promoção da Saúde, Tecnologia da Informação

## Abstract

**Objectives:**

to assess the effectiveness of a group and telephone educational intervention with seropositive women about knowledge about HIV sexual transmission prevention.

**Methods:**

a quasi-experimental before-and-after study, carried out with 151 women living with HIV in a Specialized Care Service in a Brazilian capital. The educational intervention was carried out in three moments, with the assessment being carried out before the first and after the last moment.

**Results:**

97.4% of study participants were cisgender women aged between 18 and 58 years; 55.6% considered themselves brown; and 32.5% of interviewees had elementary school. Regarding knowledge about HIV sexual transmission, in 78.5% of items, there was an association (p<0.005) with increased participants’ knowledge after receiving the intervention.

**Conclusions:**

the educational intervention helped to increase the knowledge of women living with HIV about the sexual transmission of the infection.

## RESUMO

**Objetivos:**

avaliar a efetividade de uma intervenção educativa em grupo e por telefone com mulheres soropositivas acerca do conhecimento sobre a prevenção da transmissão sexual do HIV.

**Métodos:**

estudo quase-experimental do tipo antes e depois, realizado com 151 mulheres vivendo com HIV em um Serviço de Atenção Especializada de uma capital do Brasil. A intervenção educativa foi realizada em três momentos, com a avaliação sendo feita antes do primeiro e após o último momento.

**Resultados:**

97,4% das participantes do estudo eram mulheres cisgênero e tinham idade entre 18 e 58 anos; 55,6% se consideravam pardas; e 32,5% das entrevistadas possuíam ensino fundamental. Em relação ao conhecimento sobre a transmissão sexual do HIV, em 78,5% dos itens, houve associação (p<0,005) com aumento do conhecimento das participantes após receberem a intervenção.

**Conclusões:**

a intervenção educativa colaborou para o aumento do conhecimento das mulheres vivendo com HIV sobre a transmissão sexual da infecção.

## RESUMEN

**Objetivos:**

evaluar la efectividad de una intervención educativa grupal y telefónica con mujeres seropositivas sobre conocimientos sobre la prevención de la transmisión sexual del VIH.

**Métodos:**

estudio cuasi-experimental de antes y después, realizado con 151 mujeres viviendo con VIH en un Servicio de Atención Especializada de una capital brasileña. La intervención educativa se realizó en tres momentos, realizándose la evaluación antes del primero y después del último momento.

**Resultados:**

el 97,4% de los participantes del estudio eran mujeres cisgénero con edades entre 18 y 58 años; el 55,6% se consideraba moreno; y el 32,5% de los entrevistados tenía educación primaria. En cuanto al conocimiento sobre la transmisión sexual del VIH, en el 78,5% de los ítems, hubo asociación (p<0,005) con mayor conocimiento de los participantes después de recibir la intervención.

**Conclusiones:**

la intervención educativa ayudó a aumentar el conocimiento de las mujeres que viven con VIH sobre la transmisión sexual de la infección.

## INTRODUCTION

HIV infection still represents a global health crisis, with 37.7 million people living with the infection in 2020, and of these, 20.2 million are women and girls^(1, 2)^. In Brazil, from 2007 to June 2021, 381,793 cases of HIV infection were reported in the Notifiable Diseases Information System (SINAN – *Sistema de Informação de Agravos de Notificação*), of which 115,333 were women^(3)^. Living in the context of HIV/AIDS means facing many challenges, which strongly interfere in the lives and, in particular, in the behavior of these people, and can influence the spread of the epidemic^(2)^.

Knowledge about HIV is the pillar of the fight against AIDS, by overcoming concepts, reducing stigma against people living with HIV (PLHIV), promoting positive attitudes and changes in sexual behavior^(4)^. Studies indicate that behaviors that increase the risk of HIV infection, such as inconsistent condom use during sexual intercourse, were associated with lack of knowledge about risks of infection^(5)^.

Having access to adequate information favors understanding and awareness, becoming a positive factor that favors the negotiation of sexual protection practices between sexual partners^(6)^, which minimize the risks of acquiring or transmitting the infection^(7)^.

Studies with women living with HIV (WLHIV) point to a context of moral and cultural issues, gender inequality and violence^(1)^, suggesting that vulnerabilities lead these women to difficulty accessing health services, difficulty negotiating safe sexual behavior with their partner, inadequate knowledge of HIV prevention alternatives, which leads them to risk behavior for infection^(8)^.

Thus, it is necessary for the multidisciplinary team, especially nurses, to implement educational interventions capable of expanding and modifying the level of knowledge and preventive behavior related to HIV sexual transmission in these women. For this, it is necessary to consider their living conditions, support network, situations of discrimination, gender inequality, sexual practices and prevention^(9)^.

Several educational interventions (behavioral, structural and biomedical) that act at the individual and population level have been used with the aim of preventing HIV and providing support to these individuals^(10)^. The health education model supported by technologies reinforces health professionals’ skills by expanding the possibilities of their work^(11)^, and also increases the reach of interventions and their effectiveness in relation to HIV prevention^(12)^.

Evidence points to many studies of interventions aimed at women in the general population, pregnant women, transsexuals or PLHIV, with positive results^(10, 13, 14)^. But they usually do not emphasize gender differences in vulnerability to HIV, and they do not emphasize knowledge about HIV sexual transmission.

It is necessary that interventions are designed with structural, social and behavioral approaches and that the available tools are maximized and adapted to the target population, in order to effectively promote HIV prevention and treatment.

The Health Belief Model (HBM) is a theoretical model built on the basis of psychological and social theory, which has as its premise that individuals are more predisposed to behavioral changes when they perceive their vulnerability to the disease, the consequences or its severity, the benefits with the adoption of preventive measures and whether preventive actions bring benefits that outweigh the losses involved in the adoption of these practices^(15, 16)^.

## OBJECTIVES

To assess the effectiveness of a group and telephone educational intervention with WLHIV on knowledge about preventing HIV sexual transmission.

## METHODS

### Ethical aspects

This research was approved by the Research Ethics Committee of the *Universidade de São Paulo* at *Escola de Enfermagem de Ribeirão Preto*. Subsequently, as a result of the COVID-19 pandemic, authorization was requested from the committee to continue the research in hybrid format (face-to-face and virtual). The amendment was approved and, only after approval, virtual interventions with participants began. All ethical aspects were respected.

### Study design, period and place

This is a quasi-experimental before-and-after study. The quasi-experimental study is a longitudinal study in which cause and effect relationships between independent and dependent variables are examined, with no control group^(17)^. The research followed the recommendations of the Consolidated Standards of Reporting Trials (CONSORT) statement and a checklist derived from it, the Checklist The TIDieR (Template for Intervention Description and Replication)^(18)^, which contributed to the elaboration of the intervention stages.

Data collection and intervention took place from November 2020 to August 2021, at the Specialized Care Service (SCC), a reference in the care of sexually transmitted infections and HIV/AIDS, at the Service Center (PAM) *Salgadinho*, in Maceio, capital of Alagoas.

### Population, sample, and inclusion and exclusion criteria

The study population consisted of all WLHIV, who were in outpatient follow-up at the SCC, around 614 women. The invitations to the participants were made by the researcher and her team, carried out in the waiting room, while the patients were waiting for their medical appointments. The sample selection was carried out based on inclusion and exclusion criteria, using the non-probabilistic sampling technique and consecutive sampling. The research team consisted of the main researcher, four undergraduate nursing students and two clinical nurses. The team received training to conduct data collection and received information about the instruments, appropriate ways to approach women and how to conduct interventions. Thus, the team was adequately trained for this research.

**Figure 1:**
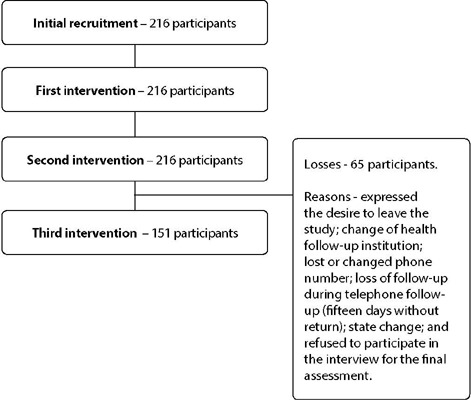
Participation of women in the intervention and composition of the final sample

To carry out the sample calculation, a difference of 20% was considered (p0 = 50% and p1 = 70%), a significance level of 5% (α = 0.05), test power of 80% (β = 0.80), correlation between measurements of 50% (ρ = 0.50). The calculated sample size was 142 participants^(19)^. Possible losses due to discontinuity of the sample due to reasons unrelated to the conduct of the study were also estimated. With this, an increase in the sample of at least 30% of the initial calculation was calculated. Initially, 216 women were included in the study, and 151 completed all stages, according to Figure 1.

The study included cisgender and transgender women aged 18 years or older, residing in the state of Alagoas, using antiret-roviral therapy (ART) for at least six months, sexually active for at least six months, being followed up at the SCC, which had a cell phone with WhatsApp®.

Women in the pregnancy-puerperal period, those with severe psychiatric disorders, those with severe hearing impairment (low acuity or deafness) or visual impairment (low visual acuity or blindness) those who were unable to go to the service due to health conditions or because they were confined, as prisoners, institutionalized and residents in support houses, were excluded. The respective exclusion criteria were detected by the researchers through observation or information from the service’s health team.

The discontinuity criteria were when participants expressed the desire to abandon the study, change of health follow-up institution, loss or change of telephone number, loss of follow-up during telephone follow-up (fifteen days without returning), change of state, and refuse to participate in the interview for final assessment.

Considering the inclusion and exclusion criteria, 216 participants were recruited, who performed the first assessment of the research. Of these, only 151 completed the intervention protocol. The percentage of loss was 30%, and the main reasons for discontinuing were changing the phone number and not wanting to participate in the last stage of the study.

### Study protocol

Data were collected through individual interviews, at the service itself, by the researcher and her team, in a private environment, guided by a data collection questionnaire adapted from another study^(20)^ and validated by face and content by experts and by the target population.

The questionnaire contained variables of a sociodemographic nature, such as age, gender, skin color, education, occupation and family income. Clinical variables were composed of treatment time, viral load status, situation of violence and comorbidities. Behavioral variables included partner type, quantity and sero-positivity, forms of prevention, frequency of condom use, knowledge of female condom, use of female condom, knowledge of pre-exposure prophylaxis (PrEP) and post-exposure prophylaxis (PEP) and disclosure of diagnosis. Knowledge variables about the risk of HIV sexual transmission included vaginal, anal and oral sex, the use of condoms in all sexual relations, the risk of sexually transmitted infections, risk of undetectable viral load, risk of using alcohol and drugs, knowledge of their partner’s seropositivity, risk of using the withdrawal of the penis before ejaculation, risk of unprotected intercourse by seropositive couple and risk of transmission when using PEP and PrEP.

The interventions initially took place in person and by sending messages via WhatsApp®. Subsequently, due to the increase in cases in the COVID-19 pandemic in Maceió-AL, there was a suspension of activities at the SCC, where the interventions of the last meeting began to occur in a hybrid way. It was only remote, through video calls via WhatsApp®, during the suspension of activities (March 1 to April 28, 2021), and remote and face-to-face, after the return of face-to-face activities (from April 28, 2021).

The educational messages used in the research intervention were adapted from another study^(21)^ and underwent a face and content validity process by experts and the target population, which included the clarity and degree of item relevance^(22)^. After validity, a percentage of 85.2% of agreement was obtained among the experts regarding message clarity, obtained through the percentage of interobserver agreement. In it, the number of elements that had a “yes” answer is divided by the total number of elements assessed, and at the end, the result of this count is multiplied by 100. The degree of relevance was 0.95 obtained through the Content Validity Index (CVI). Thus, 91.67% of women (target population) stated that the messages were good and easy to understand. These messages were sent via WhatsApp® to research participants.

The face-to-face educational intervention was applied to the participants by the researcher and her team, which was trained for this purpose. The meetings took place in groups and individually at the unit itself from November 2020 to August 2021. The same participant was assessed before the first moment and after the last moment of the intervention: (assessment 1+ I1 (intervention), I2 (intervention), I3 (intervention) + reassessment).

The intervention was divided into three moments, being applied in the period of two months. In the first meeting, there was a dynamic presentation and explanation of the methodology that would be used. Then, after signing the Informed Consent Form (ICF), anassessment questionnaire was applied (pre-test/assessment 1), and contents were presented in a 30-minute dialogued expository class, with space for questions and clarifications of doubts (intervention I1).

Educational messages were sent weekly via WhatsApp® and dealt with the same content addressed in face-to-face meetings, but were validated for this purpose and adapted to this population (intervention I2). The messages were sent a week after the first meeting, in a total of eight messages, and the last closing message was delivered a week before the second and last meeting. They were sent according to standardization, always at 17:00. It should be noted that the content of the messages did not contain the word “HIV”, to preserve the secrecy of participants’ diagnosis.

In the second and last meeting, all the content was reviewed and the women’s doubts were answered (intervention I3), and then the reassessment questionnaire was applied. The meetings lasted one hour on average. Face-to-face meetings occurred with a maximum of twelve people in each group and remotely individually.

The educational intervention was carried out remotely via WhatsApp® video call, at a date and time previously scheduled with participants. The remote form was only applied in the second and last meeting. The virtual meeting lasted around one hour. The guidelines were carried out during a video call, and the ICF and the questionnaire were made available in digital format on the Google Forms© platform by Google®. For those who had low education or difficulty with the internet, the option of the researcher was made available, with participants’ authorization, marking the acceptance in the ICF and writing down the answers in the digital questionnaire.

### Analysis of results, and statistics

The data collected from the questionnaire were entered into a spreadsheet in Excel for Windows 2016 and subsequently exported to a definitive database using the Statistical Package for the Social Sciences (SPSS) program, version 26.0^(23)^. Soon after, the data categorization stage was started, with tables being constructed for exposing the information, interpretation and discussion of the results obtained.

To assess the hypothesis of homogeneity of results before and after the data collection questionnaire, McNemar’schi-square test was used. To assess the levels of correct answers of participants’ items before and after the interventions, the frequency distribution of correct and incorrect answers for each of the questionnaire items was carried out.

All analyzes were performed using the SPSS26 and R Core Team^(24)^ software, with a significance level of 5% and a confidence level of 95%.

## RESULTS

Of the 151 women who participated in the study, the majority (97.4%) were cisgender and aged between 18 and 58 years (mean 39.1). Moreover, the majority (55.6%) considered themselves brown and more than a third (32.5%) of interviewees had completed elementary school. As for income, the majority (68.8) were unemployed, receiving from one to three minimum wages. Almost 30% of interviewees received less than the minimum wage, amounts related to the *Bolsa Família*(Brazilian Family Allowance) or the Emergency Aid, with the latter being provided by the federal government as a result of the COVID-19 pandemic (Table 1).

**Table 1:** Distribution of women living with HIV according to sociodemo-graphic characteristics, Maceió, Alagoas, Brazil, 2022 (N=151)

Variables	n	%
Age		
18-34	59	39.0
35-44	46	30.5
>45	46	30.5
Sex assigned at birth		
Female	147	97.4
Male	4	2.6
Color		
White	26	17.2
Black	29	19.2
Brown	84	55.6
Others	2	1.4
Missing	10	6.6
Education (years)		
None to 4	53	35.1
5 to 9	36	23.8
10 to 12	53	35.1
13 or more	9	6.0
Employment		
Employee	35	23.2
Unemployed	92	60.9
Self-employed	18	11.9
Others	6	4.0
Monthly family income*		
Less than minimum wage	45	29.8
From 1 to 3 minimum wages	104	68.8
More than 3 minimum wages	1	0.7
Missing	1	0.7

* *Considered the minimum wage in the amount of R$ 1,045.00 (US$190.00).*

Regarding participant clinical characteristics (Table 2), the majority (71.1%) had up to 10 years of treatment and 81 of them (53.6%) had an undetectable viral load in the last six months. It was found that almost a third (28.5%) of interviewees did not have a viral load test for the last six months in their medical records. It was identified that 60 women (39.5%) lived or are living in situations of violence, and most interviewees did not present comorbidities in the last year, with the exception of anogenital infection, which was presented by the majority (56.3%) of them.

**Table 2:** Distribution of women living with HIV according to clinical characteristics, Maceió, Alagoas, Brazil, 2022 (N=151)

Variables	n	%
Treatment time		
Up to 2 years	35	23.2
> 2 to 5 years	44	29.1
> 5 to 10 years	36	23.8
Greater than 10 years	36	23.8
Viral load in the last six months		
Detectable	22	14.6
Undetectable	81	53.6
No valid exam for the indicated period	43	28.5
Missing	5	3.3
Suffers or suffered some type of violence		
Yes	60	39.7
No	88	58.3
Does not know	2	1.3
Missing	1	0.7
Has comorbidity		
Yes	71	47.0
No	80	53.0
Had an anogenital infection in the last year		
Yes	85	56.3
No	63	41.7
Missing	3	2.0
Comorbidities		
Diabetes mellitus		
Yes	16	10.6
No	135	89.4
Hypertension		
Yes	20	13.2
No	131	86.8
Depression		
Yes	38	25.2
No	113	74.8
Triglyceridemia		
Yes	6	4.0
No	145	96.0
Other		
Yes	16	10.6
No	135	89.4

When analyzing the preventive behavior of women in relation to HIV sexual transmission, distributed in Table 3, it is noticed that 134 (88.7%) of them had a steady partner, 140 (92.7%) had around 1 to 2 partners during the year,83 (55%) had serodifferent partners, 138 (91.4%) of them use some form of HIV sexual transmission prevention and most 99 (65.5%) revealed the diagnosis to their partner.

**Table 3:** Distribution of women living with HIV according to behavioral characteristics, Maceió, Alagoas, Brazil, 2022 (N=151)

Variables	n	%
Type of partner		
Fixed	134	88.7
Casual	17	11.3
Number of sexual partners in the last year		
1 to 2	140	92.7
3 to 5	8	5.3
Above 6	3	2.0
HIV-positive partner		
Yes	46	30.5
No	83	55.0
Does not know	16	10.6
Missing	6	4.0
Uses some form to prevent HIV sexual transmission		
Yes	138	91.4
No	13	8.6
About the frequency of condom use when having sex in the last six months		
Always	88	58.3
Most of the time	8	5.3
Sometimes	19	12.6
Almost never	16	10.6
Never	20	13.2
If knows the inner condom (female)		
Yes	135	89.4
No	14	9.3
Missing	2	1.3
Used female condom		
Yes	52	34.4
No	91	60.3
Not applicable	6	4.0
Missing	2	1.3
If knows PrEP and PEP		
Yes	51	33.1
No	101	66.9
If revealed their diagnosis		
Yes	99	65.5
No	33	21.9
Other	11	7.3
Assesses whether the viral load is undetectable		
Yes	41	27.2
No	110	72.8
Withdraws the penis before ejaculation		
Yes	7	4.6
No	144	95.4
Avoids anal sex		
Yes	10	6.6
No	141	93.4
Avoids vaginal sex		
Yes	-	-
No	151	100.0
Avoids oral sex		
Yes	12	7.9
No	139	92.1
Other		
Yes	2	1.3
No	149	98.7

While these data reveal a preventive behavior for HIV, on the other hand, 88 (58.3%) interviewees used condoms consistently, and this means that, of the 151 WLHIV interviewed, 63 (41.7%) are not protecting themselves against sexually transmitted infections (STIs) or against other strains of HIV. In addition to this, they can transmit the infection to their partners, since the vast majority (97.4%) do not know about PrEP, and 110 (72.8%) of them do not assess their viral load to have sexual intercourse with their partner, according to data presented in Table 3.

When analyzing Table 4, it is noticed that there was an increase in the correct answers in all (100%) items assessed after the interventions. In most (78.5%) of items, there was an association (p<0.005) with the change in participants’ knowledge after receiving the intervention. The highest percentages of correct answers (>25%) occurred in item 12 (regarding the risk of the couple who have HIV contaminating themselves), which increased from 93 to 132 correct answers (p=0.0001), and in item 14 (how much to the risk of being contaminated using PrEP), which increased from 10 to 49 correct answers (p=0.00098).On the other hand, in the majority (69.2%) of items, the number of correct answers before the intervention was low, as in item 9, since only seven (4.6%) of interviewees stated that they did not know the partner’s serology poses a risk of virus transmission, and this value increased to 15 (9.9%) after the interventions. Regarding the number of omissions shown in Table 4, they refer to the number of participants who chose not to answer each question, and it is possible to note that they had small percentages (1.7% to 2.0%).

**Table 4:** Analysis of knowledge of women living with HIV about the risk of HIV sexual transmission before and after the intervention, Maceió, Alagoas, Brazil, 2022 (N=151)

	Before n (%)			After n (%)		*p* value
	Hits	Misses	Missing	Hits	Misses	
1. Vaginal sex without a condom carries a risk of HIV transmission.	130 (86.1%)	21 (13.9%)	-	136 (90.1%)	15 (9.9%)	0.3536
2. Anal sex without a condom poses a risk of HIV transmission.	131 (86.8%)	20 (13.2%)	-	137 (90.7%)	14 (9.3%)	0.1675
3. Oral sex (mouth on penis) carries a risk of HIV transmission.	35 (23.2%)	113 (74.8%)	3 (2.0%)	46 (30.5%)	105 (69.5%)	0.0001
4. Oral sex (mouth to vagina) carries a risk of HIV transmission.	23 (15.2%)	127 (84.1%)	1 (0.7%)	50 (33.1%)	101 (66.9%)	0.0000
5. Even using a condom every time having sex, there is still a risk of HIV transmission.	39 (25.8%)	109 (72.2)	3 (2.0%)	58 (38.4%)	93 (61.6%)	0.0192
6. The presence of STI (venereal disease) presents a risk of HIV transmission during sexual intercourse without a condom.	10 (6.6%)	140 (92.7%)	1 (0.7%)	16 (10.6%)	135 (89.4%)	0.0006
7. If the amount of virus in the body is very low (undetectable viral load), there is a risk of transmitting HIV.	28 (18.5%)	122 (80.8%)	1 (0.7%)	49 (32.5%)	102 (67.5%)	0.0002
8. Having sex under the influence of alcohol or drugs poses a risk of HIV transmission.	6 (4.0%)	145 (96.0%)	-	31 (20.5%)	120 (79.5%)	0.0004
9. Not knowing whether a partner has HIV poses a risk of transmitting the virus.	7 (4.6%)	143 (94.7%)	1 (0.7%)	15 (9.9%)	136 (90.1%)	0.2320
10. withdrawing the penis before ejaculation (cum outside) poses a risk of HIV transmission.	84 (55.6%)	67 (44.4%)	-	88 (58.3%)	63 (41.7%)	0.0080
11. When the couple has HIV and has sex without a condom, this attitude poses a risk of HIV transmission.	93 (61.6%)	56 (37.1%)	2 (1.3%)	132 (87.4%)	19 (12.6%)	0.0001
12. There is a risk of transmitting HIV when a partner who does not have HIV uses PEP.	11 (7.3%)	140 (92.7)	-	40 (26.5%)	111 (73.5%)	0.0043
13. There is a risk of transmitting HIV when a partner who does not have HIV uses PrEP.	10 (6.6%)	141 (93.4%)	-	49 (32.5%)	102 (67.5%)	0.0009

*Source: direct research. McNemar’s chi-square test was used.*

## DISCUSSION

### Characterization of women living with HIV according to sociodemographic and clinical variables

This study assessed the effectiveness of a group and telephone educational intervention with WLHIV about knowledge about preventing HIV sexual transmission. To carry out this investigation, it was necessary to build and validate educational messages to be sent to the WLHIV, as well as to use digital tools such as Google Drive, WhatsApp® video call tool, Google Forms© by Google®, Excel for Windows 2016 and SPSS26.

A study carried out in Pará found a profile of WLHIV similar to this research, pointing out that the majority (63.0%) of interviewees had low education, were unemployed (69.8%) and only 53.5% had an undetectable viral load^(25)^. As for employment situation, in this study, unemployment reached more than half of participants, which can have a negative influence on several areas of these women’s lives.

A correlational study with WLHIV in the United States compared a group of employed women with a group of unemployed women, and having a job was associated with better quality of life, better compliance with consultations and antiretrovirals, HIV viral suppression and less depressive symptoms, stress and anxiety^(26)^. As for anogenital lesions, the prevalence in this study was higher than that identified in a North American study, in which 27% of WLHIV had high-grade squamous intraepithelial lesions of the anus^(27)^. The importance of intervening actions for health promotion and STI prevention is highlighted.

Other data that drew attention were the situations of violence experienced by the research participants. Data were identified that were higher than the world index among women in general, which is 35%^(1)^. An international study shows that, in Africa, women who have HIV are more likely to experience intimate partner violence than those who do not have HIV. In these cases, violence can interfere with seeking care and treatment as well as with disclosing the diagnosis^(28)^.

### Characterization of women living with HIV according to behavioral variables

As for the type of partnership, the data from this research are in line with another study carried out with WLHIV, which showed a similar result: 75.7% of interviewees had only one sexual partner^(25)^. Another study conducted with WLHIV found that 77.0% of women had only one partner^(29)^. These data reflect a preventive behavior for HIV; however, studies indicate that the increase in HIV infection among women is associated with having steady partners, as they trust their partner^(13)^.

Disclosure of sexual partner’s diagnosis is an important factor for prevention, as it favors dialogue about sexual protection, especially for serodifferent couples. In this study, participants had serodifferent partners. In another study with PLHIV, the number of participants with serodifferent partnerships was higher than that identified in this research. It was found that 72% of respondents had serodifferent partners, and this was associated with consistent condom use^(30)^.

Behavioral characteristics and knowledge about HIV and other STIs influence prevention, such as low rates of consistent condom use and lack of knowledge about PrEP, PEP and failure to assess viral load to have sexual intercourse identified in this study. These data denounce a population at risk both for transmitting HIV and for being contaminated with other strains of the virus and other STIs.

This reality is experienced by Brazil and other countries and regions. In a cross-sectional study conducted in Atlanta with WLHIV, it showed that 53% of them reported inconsistent condom use^(31)^. In a study conducted in southern Ethiopia with PLHIV, 52.4% of whom were female, participants reported having unprotected sex in 40.9% of the study population^(32)^. These data indicate the need to implement strategies that include WLHIV in a broader prevention plan, using different methods and providing direct, clear and accessible information for people with different levels of education.

There are many factors that interfere with the use of condoms by women, and one of them is the lack of dialogue about sexual protection with their partners. A study with WLHIV revealed that 59% of women who consistently used condoms said they discussed sexual protection with their partner. Moreover, inconsistent condom use was associated with the consumption of alcohol and other drugs before sexual intercourse^(29)^.

A possible alternative to promote health for this population would be using female condom. However, although participants know the method, use compliance is not satisfactory. Strategies aimed at motivating using this condom should be implemented in health institutions, to increase women’s autonomy regarding their choices about methods for safe sex^(33)^, as women can decide on their own about the moment to use it.

### Effectiveness of educational interventions regarding the knowledge of women living with HIV about the risk of HIV sexual transmission

When analyzing the effects of the interventions regarding WLHIV’s knowledgeon the risk of HIV sexual transmission, it is noticed that there was an increase in the correct answers in all assessed items.

Knowledge about HIV sexual transmission in the general population is considered low. For women, especially WLHIV, having an adequate level of knowledge in this area means making more conscious choices and decisions about life, health, sexuality and pregnancy^(34)^.

When considering that this research deals with WLHIV and that all of them had an active sex life, access to knowledge above that of the general population about HIV sexual transmission is essential. The lack of adequate information exposes this population to the risk of vulnerability to HIV^(5, 35)^. A study with PLHIV showed that the lack of knowledge about HIV transmission and prevention leads people to be more likely (2.4 times) to have unprotected sex and that women were 1.94 times more likely to have sex without condom, compared to males^(32)^.

On the other hand, a study with WLHIV in Sweden revealed that the high level of knowledge regarding HIV reduces the consequences of infection stigmatization in their lives, influencing healthy sexual practices. This knowledge comes from several segments, including health professionals^(34)^.

In our study, there was an increase in correct answers in all items after the interventions, showing the positive change in participants’ knowledge. In the same way, an intervention study carried out among seronegative partners of HIV-positive people in Thailand associated the intervention with an improvement in the level of knowledge about HIV infection and sexual transmission prevention^(36)^.

An intervention study in the United States showed that interventions aimed at WLHIV’s knowledge increase the chances of behavioral change and promote safe sexual behavior. In particular, the intervention focused on attitudes and beliefs that support condom use was related to lower odds of unprotected sex with an HIV-positive or HIV-negative partner. The study suggests the use of interventions that promote the reduction of risk behaviors and the improvement of skills to discuss condom use^(37)^.

On the other hand, our study showed a low number of correct answers for the items before the interventions, especially in questions related to the risk of transmitting HIV, when the viral load is low (undetectable), when people use the recommended antiretrovirals and when using PrEP or PEP. This fact points to a low level of knowledge on this topic, which shows the need for intervention aimed at this population.

Regarding interventions with sending messages through digital means, several studies have reported this type of strategy fortreatment compliance, in addition to the management and prevention of various diseases^(38, 39, 40)^. An international study shows that sending messages to PLHIV is very promising to reinforce health promotion actions, such as ART compliance^(41)^.In a review, it was identified that text messages sent through cell phones are effective in improving treatment compliance among PLHIV^(42)^. Given this, it is clear that this type of intervention is an important contribution to health promotion and WLHIV in Brazil.

### Study limitations

In this study, there were some limitations, such as the possibility of social desirability bias related to stigma and prejudice, which permeate the questions listed in the research, in which participants can respond according to norms or in a socially acceptable manner. Another point is that the study dealt with revelations about women’s intimate life, which caused embarrassment for some. To minimize these effects, the researchers were all women and tried to make the interviewees feel at ease, calm and in a private environment. Additionally, the objectives of the study were explained, seeking to conduct data collection in an environment of respect, free of judgments and guaranteeing the confidentiality of the information obtained.

### Contributions to nursing

Our research carried out educational interventions in groups and over the telephone and showed promising results, as it changed WLHIV’s knowledge, enabling the expansion of their perception regarding the risks of HIV sexual transmission arising from their sexual behavior. Therefore, we emphasize that its use is recommended to enhance nurses’ professional performance, since, as a professional educator, nurses must offer ways to increase the knowledge of this population and modify negative beliefs that permeate the infection, to enable decision-making aimed at HIV-preventive behavior. In this way, using interventions by digital means by nurses favors disseminating knowledge, strengthening professional performance and bonding with the population.

## CONCLUSIONS

Carrying out this research made it possible to carry out educational interventions in groups and by mobile phone, via current applications, with WLHIV about knowledge about HIV sexual transmission prevention as well as assessing its effectiveness.

The study showed that it was possible to assess the effectiveness of the educational intervention, as the same group was assessed before and after receiving the intervention, achieving the objective of the research. In this way, it was observed that the intervention collaborated to increase participants’ knowledge about HIV sexual transmission prevention. Moreover, the study revealed that the majority of the investigated population has a low level of knowledge about the subject and a significant percentage of this population lives in a situation of violence, which denounces the need for interventions aimed specifically at this population.
